# Efficacy and safety of Argatroban in patients with acute ischemic stroke: a systematic review and meta-analysis

**DOI:** 10.3389/fneur.2024.1364895

**Published:** 2024-02-19

**Authors:** YiRan Cheng, ChangNing Liu, ShanShan Li, Miao Miao Meng, He Li

**Affiliations:** ^1^Affiliated Hospital of Shandong University of Traditional Chinese Medicine, Jinan, China; ^2^Neurology Department of First Clinical Medical College, Shandong University of Traditional Chinese Medicine, Jinan, China; ^3^Nanjing University of Chinese Medicine, Nanjing, China

**Keywords:** acute ischemic stroke, Argatroban, early neurological deterioration, meta-analysis, systematic review, stroke

## Abstract

**Objective:**

Argatroban is a highly promising drug for the treatment of acute ischemic stroke (AIS), but there is currently insufficient strong evidence regarding the efficacy and safety of using Argatroban in the treatment of AIS. Therefore, we conducted a systematic review and meta-analysis to evaluate the effectiveness and safety of Argatroban in the treatment of AIS.

**Methods:**

Articles on PubMed, Embase and the Cochrane Library databases were searched from these websites’ inceptions to 2th February 2023. Randomized controlled trials and observational studies on Argatroban therapy for acute ischemic stroke were included. Meta-analyses were conducted using a random-effects model.

**Results:**

Fourteen studies involving 10,315 patients were included in the meta-analysis. The results showed a significant reduction in the rate of early neurological deterioration (END) in the Argatroban group compared with the control group (OR = 0.47, 95% CI: 0.31–0.73, *I^2^* = 15.17%). The rates of adverse events were no significant difference between the two groups (ICH: OR = 1.02, 95% CI: 0.68–1.51, *I^2^* = 0.00%; major extracranial bleeding: OR = 1.22, 95% CI: 1.01–1.48, *I^2^* = 0.00%; mortality: OR = 1.16, 95% CI: 0.84–1.59, *I^2^* = 0.00%). However, the rates of mRS score of 0–1 (OR = 1.38, 95% CI: 0.71–2.67, *I^2^* = 77.56%) and mRS score of 0–2 (OR = 1.18, 95% CI: 0.98–1.42, *I^2^* = 0.00%) during the 90 days did not significantly improved in the Argatroban group. Subgroup analyses showed that the rate of END (OR = 0.41, 95% CI: 0.26–0.65, *I^2^* = 2.77%) and mRS score of 0–2 (OR = 1.38, 95% CI: 1.06–1.81, *I^2^* = 0.00%) had significantly improved when the intervention group adopted Argatroban plus Antiplatelet.

**Conclusion:**

Argatroban can improve neurological deterioration, with a low incidence of adverse events such as bleeding and death, and general analysis showed no improvement in mRS. However, subgroup analysis suggests that compared to mono-antiplatelet therapy, combination therapy of Argatroban combined with antiplatelet therapy significantly reduced the incidence of END and improved mRS scores. After using Argatroban, there was no increase in the risk and mortality of intracranial hemorrhage and other bleeding sites.

## 1 Introduction

Stroke, as the second leading cause of death and the third leading cause (1) of disability worldwide, has gradually become a major disease that cannot be ignored due to its younger onset age. Acute ischemic stroke (AIS) accounts for 80% of stroke, and approximately one-third of AIS patients experience. Early neurological deterioration (END) (2) within 1 week of onset. END is a severe complication of ischemic stroke that can lead to adverse functional outcomes, its high disability and mortality rates may place heavy burdens on families and clinical work (3). Although treatments such as intravenous thrombolysis and intravascular therapy can reduce mortality and improve patient prognosis to some extent, studies had shown that only about 30% of AIS patients had achieved vascular recanalization through intravenous thrombolysis (4). Meanwhile, about 40–50% of patients with large artery occlusion have not achieved reperfusion after bridging thrombolysis (combining intravenous thrombolysis and mechanical thrombectomy) (5, 6). It can be seen that many AIS patients still suffer END after being treated with intravenous thrombolysis. Therefore, there is an urgent need for an effective and simple method to promote vascular recanalization, prevent reocclusion, and relieve disability caused by AIS.

Argatroban is a direct thrombin inhibitor. Research shows that it can effectively relieve AIS patients’ neurological deficits and improve their daily living abilities, as well as prevent and treat reocclusion after venous thrombosis with a low risk of bleeding, thus, it is considered as a highly potential AIS treatment drug (7). However, there is currently a lack of strong evidence regarding the efficacy and safety of Argatroban in AIS patients. Disputes regarding the efficacy of Argatroban in the clinical treatment of AIS still exists among clinicians. What’s more, the approved indications of Argatroban formulations also vary in different countries (8). For AIS patients, there is still no consensus on the use and effectiveness of Argatroban, and the following issues remain unresolved: (1) Can Argatroban bring better functional improvement and prevent END for stroke patients? (2) What is the optimal usage plan for Argatroban? To treat AIS with Argatroban alone or antiplatelet drugs combined with Argatroban? Are these plans superior to simple treatment methods such as intravenous thrombolysis, antiplatelet therapy, and mechanical thrombectomy? (3) Does the use of Argatroban increase the risk of bleeding and death? To address the above questions and promote the more rational and standardized use of Argatroban by clinicians, we conducted this meta-analysis to confirm whether Argatroban has advantages in preventing END, improving neurological deficit, and reducing bleeding risk. So that it can provide more evidence for clinical use of Argatroban and improve more AIS patients’ prognosis.

## 2 Methods

The systematic review and meta-analysis was conducted according to the Preferred Reporting Items for Systematic Reviews and Meta-Analyses (PRISMA) guidelines (9). We registered the study protocol in PROSPERO (registration number CRD42023448518).

### 2.1 Data sources and search strategy

Articles on PubMed, Embase and the Cochrane Library databases were searched from these websites’ inceptions to 2th February 2023. The search strategy included the following terms related to “stroke” and “Argatroban.” The search strategy was presented in Supplementary Table S1.

### 2.2 Inclusion and exclusion criteria

Studies were considered eligible if they met the following criteria:

(1) study types included randomized controlled trials (RCTs) and observational studies; (2) participants diagnosed as AIS aged ≥18 years; (3) intervention: Argatroban (monotherapy or combination therapy); comparison: alteplase, mono antiplatelet therapy (MAPT), dual antiplatelet therapy (DAPT), mechanical thrombectomy (MT), et al.; (4) outcome was reported at least the one following indicators: END, defined as s an increase in NIHSS score of greater than or equal to 2 whitin 7 days, compared with baseline, good functional outcome at 90 days (defined as mRS score of 0–1 and mRS score of 0–2 at 90 days), intracranial hemorrhage (included symptomatic intracranial hemorrhage and asymptomatic intracranial hemorrhage), major extracranial bleeding (included gastrointestinal bleeding, skin bleeding, mucous membrane bleeding, urine bleeding, gingival bleeding, a decrease in hemoglobin of ≥2 g/dL or transfusion of ≥2 U of blood), and mortality.

Studies were excluded if they met the following criteria:

(1) Repeated studies were published; (2) full texts were not available; (3) date could not be obtained.

### 2.3 Study selection

Titles and abstracts were reviewed by two independent reviewers. Subsequently, full texts of potentially eligible studies were screened by the same reviewers. Any disagreements between the two reviewers were resolved by consulting with the third reviewer.

### 2.4 Data extraction

Data were extracted into a predetermined extraction table independently by two reviewers. Disagreement was resolved by consulting a third reviewers. The following data were extracted: author, publication year, country, study design, sample size, age, the rate of male, study intervention and comparison, follow-up duration, outcomes (continuous data were presented as mean, standard deviation, and total participants per group; dichotomous data were presented as number of events and non-events per group).

### 2.5 Quality assessment

The methodological quality of included studies was evaluated independently by two reviewers and any disagreement was resolved by consulting a third reviewers. The quality of RCTs was assessed using the Cochrane Risk of Bias tool. The tool contained six domains (seven items) and each item was evaluated in three categories: unclear bias, low risk of bias and high risk of bias. The quality of observational studies was assessed using the Newcastle-Ottawa Scale (NOS) (10). It consisted of three domains: selection of exposure, comparability, and assessment of outcome. The range of score was zero stars up to nine stars. If the score of studies was seven or more stars, the quality was considered as high quality.

### 2.6 Statistical analysis

The meta-analysis was conducted using the STATA version 17.0. Dichotomous data were pooled as the odds ratios (OR) with 95% confidence intervals (CI), while continuous data were calculated as weighted mean differences (WMD) with 95% CI. A random-effects model was used to pool effect sizes. *p* < 0.05 was considered statistically significant. Statistical heterogeneity was evaluated using Cochran’s Q test and *I^2^* statistics. The heterogeneity was considered significant with *I^2^* > 50%. If heterogeneity was detected, subgroup analyses were performed based on region, study type and study intervention. A sensitivity analysis was performed using the leave-one-out method. The publication bias was evaluated by Egger’s test for at least 10 studies.

## 3 Results

### 3.1 Search results

**Figure 1:**
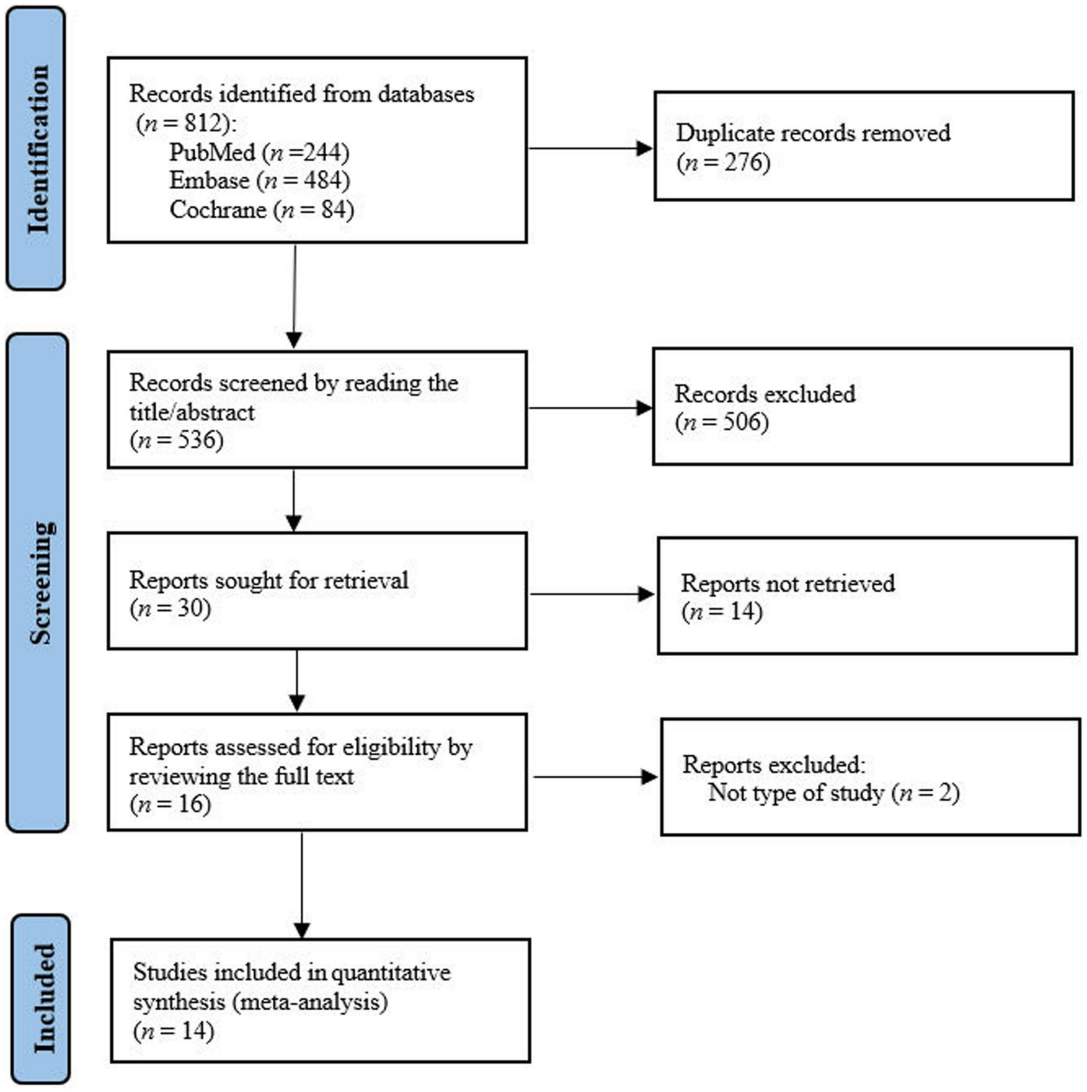
Flow chart for literature search, study selection, and reasons for exclusion.

A total of 812 studies were identified from the PubMed, Embase and Cochrane Library databases. After reviewing the full text, we found fourteenth studies were eligible for meta-analysis (11–21) (Figure 1).

### 3.2 Study characteristics

The included studies were from different countries: eight from China (11–16, 19, 22), two from Japan (18, 21), one from Korea (17), two from the USA (20, 23, 24) and one from the UK (20). Among the included studies, six studies were RCTs (11, 14, 16, 20, 22, 24) and eight studies were observational studies (12, 13, 15, 17–19, 21, 23). In the intervention group, four studies adopted Argatroban alone (16, 18, 21, 24), six studies adopted Argatroban combined with MAPT or DAPT (12–15, 19, 22), three studies adopted Argatroban combined with alteplase (11, 20, 23) and one study adopted Argatroban combined with MT (17). The characteristics of studies were shown in Table 1.

**Table 1:** Characteristics of included studies.

No.	Study	Country	Study design	Intervention group				Control group				Outcomes
				Sample size	Age	Male (%)	Interventions	Sample size	Age	Male (%)	Interventions	
1	Chen H. (11)	China	RCT	364	66 (58–72)	68.4	Argatroban alteplase	396	64 (56–71)	73.0	Alteplase	mRS score of 0–1 and mRS score of 0–2 at 90 days, END, ICH and major extracranial bleeding
2	Wang P. F. (12)	China	Observational study	136	63.0 ± 7.4	58.3	Argatroban MAPT	168	62.0 ± 7.8	57.1	DAPT	mRS score of 0–1 at 90 days, END, ICH and major extracranial bleeding
3	Chen S. (15)	China	Observational study	519	63.5 ± 10.1	68.0	Argatroban MAPT or DAPT	806	63.9 ± 11.7	66.4	MAPT or DAPT	END, mRS score of 0–2 at 90 days, ICH, major extracranial bleeding and mortality
4	Liu S (14)	China	RCT	30	57.7 ± 8.8	77.0	Argatroban MAPT or DAPT	30	57.1 ± 10.7	80.0	MAPT or DAPT	mRS score of 0–1 at 90 days, ICH, major extracranial bleeding and mortality
5	Zhou L. S. (13)	China	Observational study	35	61.7 ± 9.7	74.3	Argatroban DAPT	467	61.2 ± 12.2	73.7	DAPT	END, ICH and major extracranial bleeding
6	Kim (17)	Korea	Observational study	182	69.8 ± 13.4	52.2	Argatroban MT	120	72.7 ± 16.4	54.1	MT	mRS score of 0–2 at 90 days, ICH, major extracranial bleeding and mortality
7	Wang X. J. (16)	China	RCT	40	66.3 ± 7.9	57.5	Argatroban	40	65.1 ± 8.1	70.0	Aspirin	NIHSS, major extracranial bleeding and mortality
8	Chen L. (19)	China	Observational study	434	65.54 ± 10.83	65.2	Argatroban MAPT	1,051	65.14 ± 11.17	67.6	Aspirin	NIHSS, ICH
9	Oguro (18)	Japan	Observational study	353	76(66–83)	59.5	Argatroban	160	74(65–82)	61.9	Ozagrel	NIHSS, ICH and major extracranial bleeding
10	Barreto (20)	USA, UK	RCT	61	/	59.0	Argatroban alteplase	29	69 ± 15	54.1	Alteplase	mRS score of 0–1 at 90 days, NIHSS, ICH, major extracranial bleeding and mortality
11	Wada (21)	Japan	Observational study	2,289	/	63.7	Argatroban	2,289	/	63.0	None	mortality
12	Liu MC (22)	China	RCT	31	59.19 ± 10.83	76.0	Argatroban DAPT	36	59.13 ± 12.01	82.1	DAPT	mRS score of 0–1 and mRS score of 0–2 at 90 days, ICH
13	Sugg (23)	USA	Observational study	15	61 ± 13	66.7	Argatroban alteplase	63	/	/	Alteplase	ICH and mortality
14	LaMonte (24)	USA	RCT	117	/	41.9	Argatroban	54	65 ± 13	69.0	Placebo	mRS score of 0–2 at 90 days, ICH, major extracranial bleeding and mortality

DAPT, dual antiplatelet therapy; MAPT, mono antiplatelet therapy; MT, mechanical thrombectomy; ICH, intracranial hemorrhage.

### 3.3 Quality assessment

The quality evaluation of included RCTs was presented in Table 2. Of the six RCTs included, four trials were judged at low risk of bias (11, 14, 20, 24). Another two were considered low risk in the incomplete outcome data, selective reporting and overall risk of bias (16, 22).

**Table 2:** Quality evaluation of included RCTs.

No.	Study	Selection bias		Performance bias	Detection bias	Attrition bias	Reporting bias	Other
		Random sequence generation	Allocation concealment	Blinding of participants and personnel	Blinding of outcome assessment	Incomplete outcome data	Selective reporting	Overall risk of bias
1	Chen H. (2023)	Low	Low	Low	Low	Low	Low	Low
2	Liu S. (2020)	Low	Low	Low	Low	Low	Low	Low
3	Wang X. J. (2019)	Unclear	Unclear	Unclear	Unclear	Low	Low	Low
4	Barreto (2017)	Low	Low	Low	Low	Low	Low	Low
5	Liu M. C. (2015)	Low	Unclear	Unclear	Unclear	Low	Low	Low
6	LaMonte (2004)	Low	Low	Low	Low	Low	Low	Low

The quality evaluation of included observational studies was presented in Table 3. The eight observational studies scores seven or more stars and are also with high quality (12, 13, 15, 17–19, 21, 23).

**Table 3:** Quality evaluation of included observational studies.

No.	Study	Selection				Comparability	Exposure			Sum
		1	2	3	4	5	6	7	8	
1	Wang P. F. (2021)	1	1	0	1	1	1	1	1	7
2	Chen S. (2020)	1	0	0	1	2	1	1	1	7
3	Zhou L. S. (2020)	1	1	0	1	2	1	1	1	8
4	Kim (2019)	1	0	0	1	2	1	1	1	7
5	Chen L. (2018)	1	1	0	1	2	1	1	1	8
6	Oguro (2018)	1	1	0	1	1	1	1	1	7
7	Wada (2016)	1	1	0	1	1	1	1	1	7
8	Sugg (2006)	1	1	0	1	1	1	1	1	7

1-Is the case definition adequate; 2-Representativeness of the cases; 3-Selection of controls; 4-Definition of controls; 5-Comparability of cases and controls on the basis of the design or analysis; 6-Ascertainment of exposure; 7-Same method of ascertainment for cases and controls; 8-Non-response rate.

## 4. Results of meta-analyses

4

### 4.1 Neurologic function

**Figure 2:**
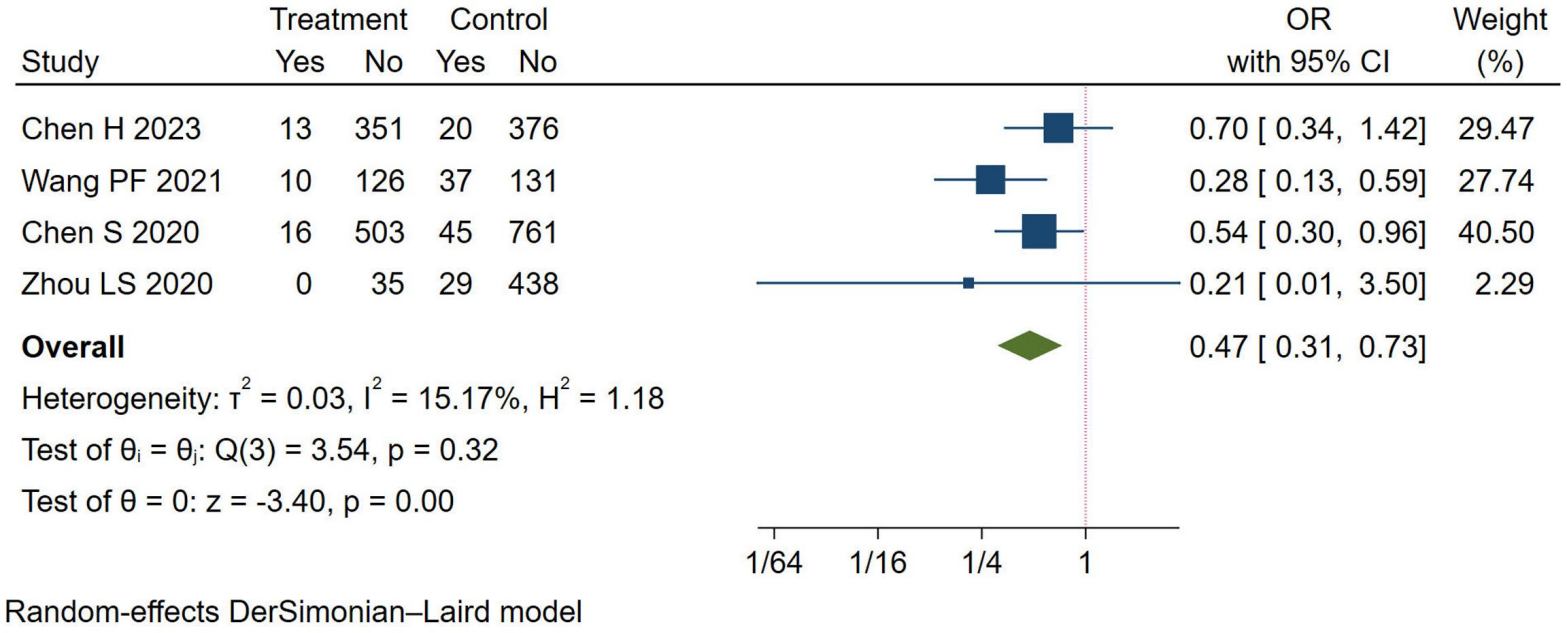
Meta-analysis of early neurological deterioration (END) in the Argatroban group compared with the control group.

END was used for evaluating neurologic function. A total of 4 studies reported the rate of END (11–13, 15). Compared with the control group, the results revealed a significant reduction in END in the Argatroban group (OR = 0.47, 95% CI: 0.31–0.73, *I^2^* = 15.17%, Figure 2). Subgroup analyses were conducted in terms of the region, study type and study intervention (Table 4), which showed that the rate of END decreased significantly only when the study design was observational study, and when the intervention group adopted Argatroban plus antiplatelet (OR = 0.41, 95% CI: 0.26–0.65, *I^2^* = 2.77%).

**Table 4:** Subgroup analysis of the END, mRS and adverse events.

	Subgroup	Number of studies	OR (95% CI)	*I^2^* (%)
*END*				
Region	Asian	4	0.47 (0.31–0.73)	15.17%
	non-Asian	0	/	/
Study type	RCT	1	0.70 (0.34–1.42)	/
	Observational study	3	0.41 (0.26–0.65)	2.77%
Study intervention	Argatroban	0	/	/
	Argatroban + alteplase	1	0.70 (0.34–1.42)	/
	Argatroban + antiplatelet	3	0.41 (0.26–0.65)	2.77%
	Argatroban + MT	0	/	/
*mRS score of 0–1 at 90 days*				
Region	Asian	4	1.31 (0.60–2.86)	82.90%
	Non-Asian	1	1.73 (0.61–4.95)	/
Study type	RCT	4	0.99 (0.74–1.30)	0.00%
	Observational study	1	3.17 (1.95–5.18)	/
Study intervention	Argatroban	0	/	/
	Argatroban + alteplase	2	1.03 (0.70–1.53)	11.95%
	Argatroban + antiplatelet	3	1.48 (0.53–4.13)	71.76%
	Argatroban + MT	0	/	/
*mRS score of 0–2 at 90 days*				
Region	Asian	4	1.22 (1.01–1.48)	0.00%
	non-Asian	1	0.77 (0.38–1.56)	/
Study type	RCT	3	0.95 (0.70–1.28)	0.00%
	Observational study	2	1.35 (1.07–1.71)	0.00%
Study intervention	Argatroban	1	0.77 (0.38–1.56)	/
	Argatroban + alteplase	1	0.98 (0.69–1.39)	/
	Argatroban + antiplatelet	2	1.38 (1.06–1.81)	0.00%
	Argatroban + MT	1	1.22 (0.77–1.94)	/
*ICH*				
Region	Asian	9	1.03 (0.64–1.66)	0.00%
	Non-Asian	3	1.31 (0.40–4.25)	52.43%
Study type	RCT	5	0.94 (0.51–1.73)	0.00%
	Observational study	7	1.08 (0.64–1.82)	0.00%
Study intervention	Argatroban	2	2.40 (0.60–9.57)	0.00%
	Argatroban + alteplase	3	0.98 (0.43–2.24)	32.78%
	Argatroban + antiplatelet	6	1.02 (0.57–1.82)	0.00%
	Argatroban + MT	1	0.60 (0.11–3.14)	/
*Major extracranial bleeding*				
Region	Asian	8	1.07 (0.76–1.50)	0.00%
	Non-Asian	2	1.84 (0.57–6.00)	14.68%
Study type	RCT	5	1.58 (0.90–2.78)	0.00%
	Observational study	5	1.00 (0.68–1.47)	0.00%
Study intervention	Argatroban	3	2.20 (0.93–5.22)	0.00%
	Argatroban + alteplase	2	1.22 (0.58–2.56)	0.00%
	Argatroban + antiplatelet	4	1.22 (0.71–2.10)	0.00%
	Argatroban + MT	1	0.82 (0.47–1.41)	/
*Mortality*				
Region	Asian	4	0.78 (0.43–1.42)	0.00%
	Non-Asian	3	1.48 (0.51–4.33)	36.52%
Study type	RCT	3	1.21 (0.55–2.63)	0.00%
	Observational study	4	0.85 (0.47–1.56)	0.88%
Study intervention	Argatroban	2	1.22 (0.60–2.47)	0.00%
	Argatroban + alteplase	2	2.05 (0.13–31.28)	62.64%
	Argatroban + antiplatelet	2	0.61 (0.09–4.34)	0.00%
	Argatroban + MT	1	0.74 (0.32–1.70)	/

### 4.2 Good functional outcome

**Figure 3:**
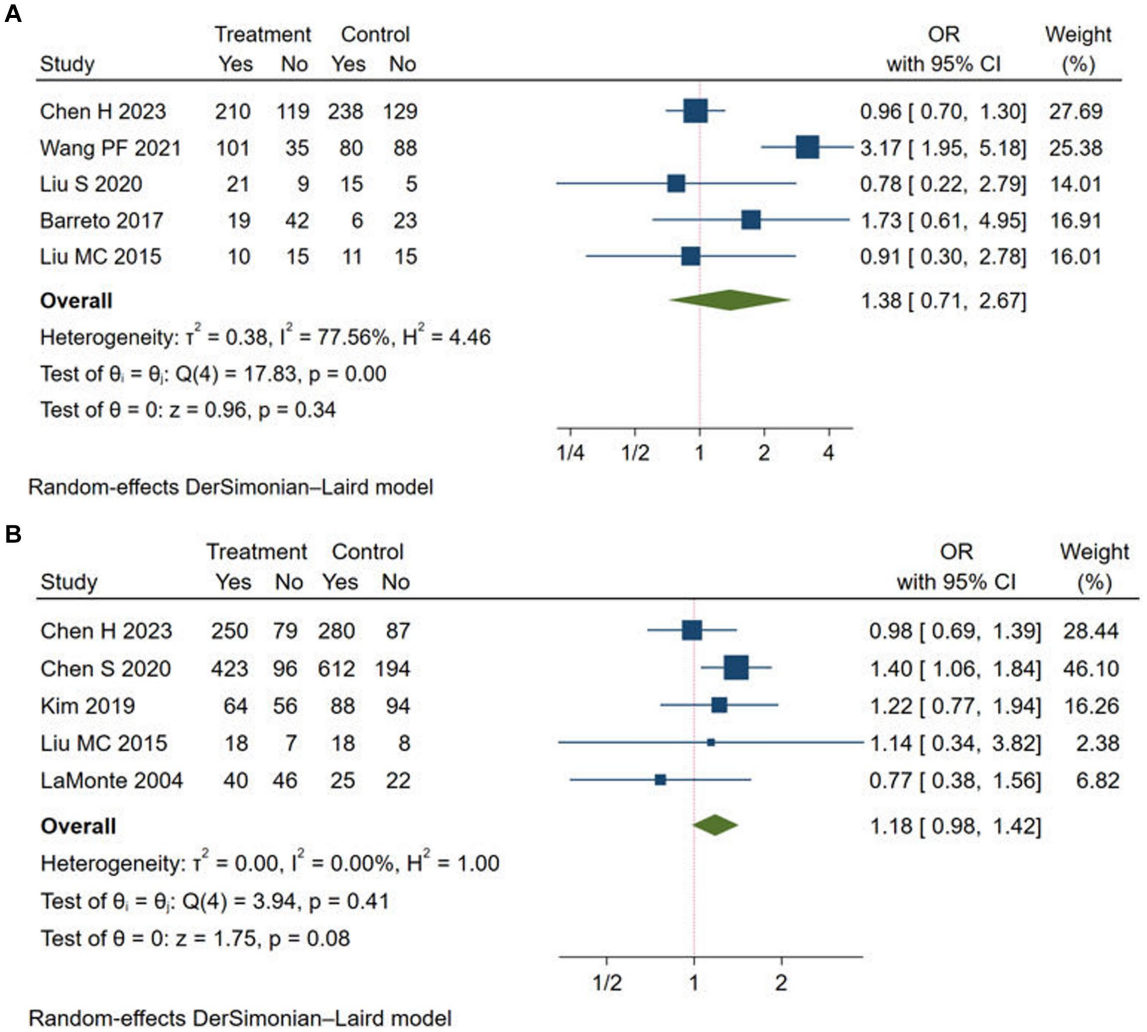
Meta-analysis of good functional outcome in the Argatroban group compared with control group. **(A)** mRS score of 0-1 at 90 days; **(B)** mRS score of 0-2 at 90 days.

We chose mRS score of 0–1 and mRS score of 0–2 at 90 days to evaluate the good functional outcome after stroke. Five studies reported the rate of mRS score of 0–1 (11, 12, 14, 20, 22) and mRS score of 0–2 at 90 days (11, 15, 17, 22, 24), respectively. The rates of mRS score of 0–1 (OR = 1.38, 95% CI: 0.71–2.67, *I^2^* = 77.56%) and mRS score of 0–2 (OR = 1.18, 95% CI: 0.98–1.42, *I^2^* = 0.00%) at 90 days did not significantly improve in the Argatroban group compared with the control group (Figure 3).

However, subgroup analyses (Table 4) suggested that the rate of mRS score of 0–1 increased in the Argatroban group when the study type was observational study (OR = 3.17, 95% CI: 1.95–5.18). Moreover, when the study type was RCT, and when the intervention group adopted Argatroban plus alteplase, the meta-analyses were quite homogeneous. The results showed that the study design and intervention might be source of heterogeneity. For the rate of mRS score of 0–2, the Argatroban group had a significant improvement compared with the control group when the participants were from Asia (OR = 1.22, 95% CI: 1.01–1.48, *I^2^* = 0.00%), when the study design was observational study (OR = 1.35, 95% CI: 1.07–1.71, *I^2^* = 0.00%), and when the intervention group adopted Argatroban plus antiplatelet (OR = 1.38, 95% CI: 1.06–1.81, *I^2^* = 0.00%). Detailed results of the analyses are shown in Table 4.

### 4.3 Adverse events

**Figure 4:**
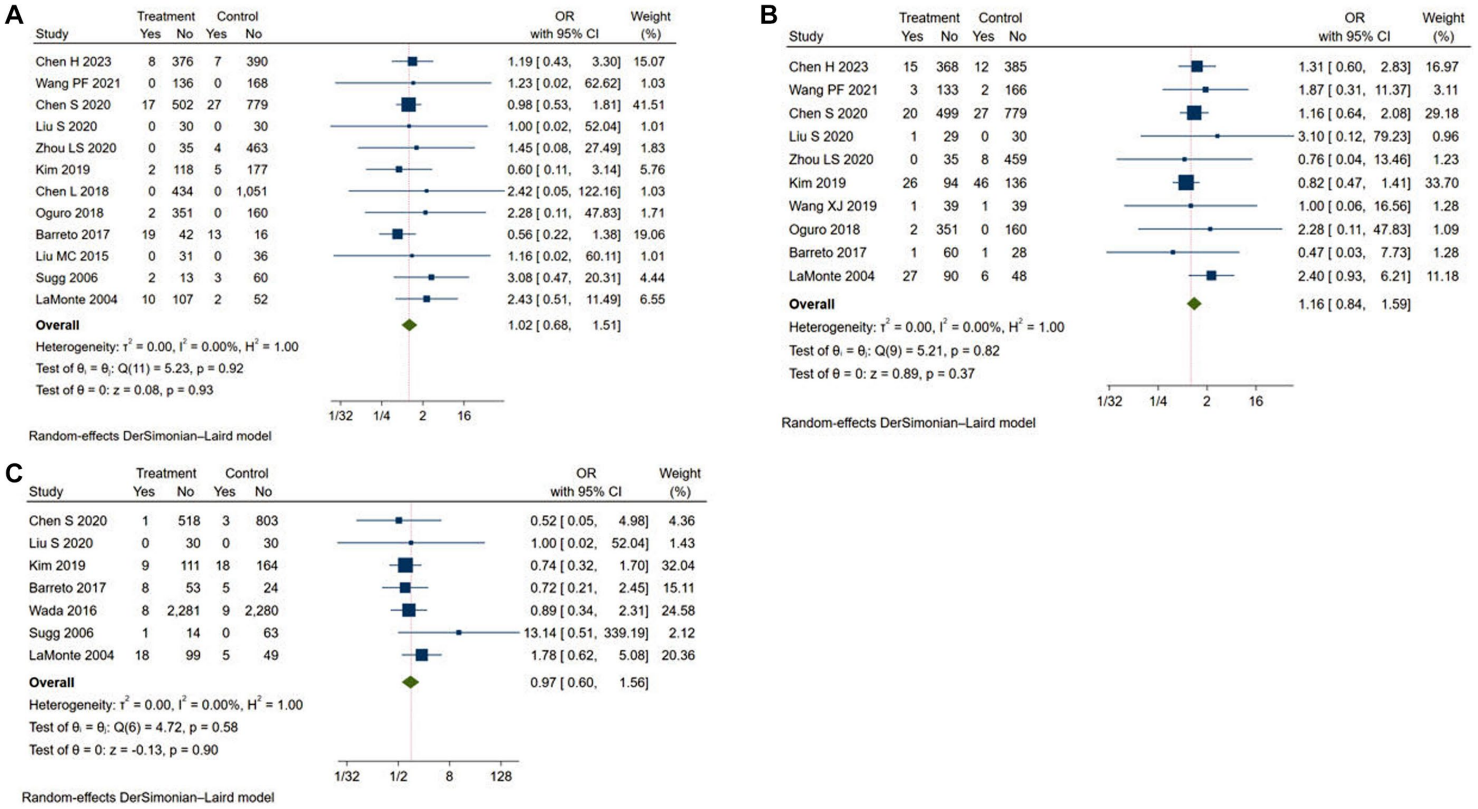
Meta-analysis of adverse events in the Argatroban group compared with control group. **(A)** intracranial hemorrhage; **(B)** major extracranial bleeding; **(C)** mortality.

In this study, adverse events included ICH, major extracranial bleeding and mortality. A total of 12 studies reported the rate of ICH (11–15, 17–20, 22, 23), 10 studies reported the rate of major extracranial bleeding (11–18, 20, 24) and 7 studies reported the rate of mortality (14, 15, 17, 20, 21, 23, 24). As shown in Figure 4, the adverse event rates of Argatroban group were no significantly different from that of the control group (ICH: OR = 1.02, 95% CI: 0.68–1.51, *I^2^* = 0.00%; major extracranial bleeding: OR = 1.22, 95% CI: 1.01–1.48, *I^2^* = 0.00%; mortality: OR = 1.16, 95% CI: 0.84–1.59, *I^2^* = 0.00%). The results of subgroup analyses showed that there were not significant differences between the different groups (Table 4).

### 4.4 Sensitivity analysis and publication bias

We performed a sensitivity analysis by omitting each study in turn (Supplementary Figures S1, S2). The results showed that pooled effect did not vary after excluding any single study. Furthermore, egger’s test did not reveal any publication bias in the analysis of the rate of ICH (*p* > |t| = 0.373).

## 5 Discussion

The results of this study suggested that Argatroban could improve neurological deterioration in AIS patients, with a low incidence of adverse events such as bleeding and death, and no improvement in mRS. Subgroup analysis suggested that compared to using antiplatelet alone, the incidence of END was significantly reduced when treated with agatroban and antiplatelet therapy, meanwhile, the mRS score significantly improved. In addition, there was no increase in mortality and the risk of intracranial hemorrhage and other bleeding sites after the use of Argatroban. In summary, the above results indicate that the combination of Argatroban and antiplatelet therapy can bring better functional improvement and prevent END in stroke patients in a safer way. It is considered as a potential therapeutic drug for ischemic cerebrovascular disease.

This study is different from the previous meta-analysis on Argatroban (25, 26) in the following three aspects. Firstly, there were a relatively small number of clinical trials involved in previous meta-analysis, ranging from 4 to 11, while a total of 14 clinical trials were included in this study. Secondly, most of the clinical trials included in previous studies were retrospective and non-blind studies. This study included more large-scale double-blind randomized controlled trials and high-quality researches in recent years. Thirdly, this study concluded through subgroup analysis that the combination of Argatroban and antiplatelet drugs was the most effective and safe treatment for ischemic stroke, which has certain guiding value for clinical practice.

Agatroban is a direct thrombin inhibitor that selectively inhibits thrombin, thereby inhibiting thrombin induced fibrinogen formation, platelet aggregation, and vasoconstriction (27). In addition, Argatroban also has the functions of vasodilation, microcirculation improvement, and potential of antivirus (28). In patients with progressive cerebral infarction, there is a significant increase in coagulation and fibrinolytic activity, indicating that in addition to antiplatelet drugs, anticoagulants also have great potential for preventing and treating END (29). The results of this study indicated that the combination of Argatroban and antiplatelet drugs could effectively reduce the occurrence of END in patients, improve neurological damage, and meanwhile, do not increase the risk of bleeding and mortality. All these reveals that the therapeutic effect of Argatroban on cerebral infarction is worth further promotion and research. An interesting conclusion in the results worth mention is that there is no statistical difference in the incidence of END and the improvement of neurological function between Argatroban combined with thrombolytic therapy and thrombolytic therapy alone. This is similar to the research results published in JAMA in 2023 (11). The negative results may be explained that after intravenous thrombolysis, some of the included patients had already achieved recanalization of blood vessels and maximum improvement in neurological function. Under this premise, the benefits of adding Argatroban became less. Since Argatroban can inhibit thrombin, thereby promoting the production of endogenous plasminogen activators, it can prevent further extension of the thrombus, and theoretically improve the prognosis of patients with poor thrombolysis efficacy. The latest study indicates that for patients with AIS who experience END within 48 h of onset, the good prognosis rate of combination therapy (Argatroban combined with antiplatelet drug) at 90 days is significantly higher than that of the monotherapy (use antiplatelet drug only) (30). This improvement can be achieved without increasing the risk of additional intracranial hemorrhage. Among them, 17.5% of patients in the Argatroban group received intravenous thrombolysis. This suggests that Argatroban may be more appropriate for patients who experience END after thrombolysis, as opposed to those who experience reperfusion following thrombolysis. Therefore, it is necessary to explore whether adding Argatroban to patients with poor prognosis after thrombolytic therapy can improve patients’ situation. In addition, previous studies showed that Argatroban had a better effect on large artery occlusion-type cerebral infarction (20, 31), while this study also included clinical trials with small artery occlusion-type cerebral infarction as the subject population, which may affect the research results to some extent. Although the results of the experiment were neutral, these studies indicated that it was safe and feasible to receive intravenous thrombolysis combined with Argatroban treatment. For some patients with a high probability of developing END and those who have been evaluated and predicted to be insensitive to thrombolytic therapy and not suitable for interventional therapy, the combination of Argatroban and thrombolytic therapy may still improve patient prognosis, which requires further confirmation through clinical trials with a larger and more detailed subject division. However, due to the fact that most stroke centers do not conduct vascular imaging examinations before intravenous thrombolysis in order to shorten door to needle time (DNT), it remains challenging to limit the subject of different types of vascular lesions in future prospective clinical trials.

The increased risk of bleeding associated with the concurrent use of Argatroban is a concern for most clinicians. It is well known that combining anticoagulants and antiplatelet drugs increases the risk of bleeding (32, 33). In this research, Argatroban was used in combination with aspirin and clopidogrel in all patients, but there was no increase in the risk of intracranial or other site-specific bleeding, nor an increase in mortality rates. A study in Japan (34) also reviewed and analyzed data, indicating no occurrence of symptomatic intracranial hemorrhage among subjects receiving Argatroban in combination with cilostazol and clopidogrel. Therefore, the combined use of Argatroban and antiplatelet drugs was considered safe and worthy of clinical promotion. However, it is still recommended to assess patients’ bleeding risk, as for those with lower bleeding risk, the combination of Argatroban and antiplatelet drugs may provide them with the maximum benefit. Furthermore, a sub-analysis of the study results revealed that Argatroban exhibited superior mRS improvement effects among Asian. Several studies have demonstrated that the prevalence of intracranial atherosclerosis (ICAS) is higher in Asian populations than in Western populations (35). Therefore, additional research is necessary to ascertain whether these factors impact the effectiveness of Argatroban.

There are several limitations in this study. Firstly, this study was unable to perform subgroup analysis on stroke classification. Previous studies showed inconsistent efficacy of Argatroban treatment in large artery atherosclerotic stroke and penetrating artery atherosclerosis-related stroke. The effectiveness of Argatroban in treating strokes may be related to the type of stroke, but different studies have reported conflicting findings. Additionally, existing research lacks differentiation in terms of using timing and dosage, with significant variations observed among different studies. Therefore, future large-scale clinical trials are needed to explore the effects of Argatroban on different stroke types, various dosages, and optimal timing.

## 6 Conclusion

Argatroban can improve neurological deterioration, with a low incidence of adverse events such as bleeding and death, and no improvement in mRS. Subgroup analysis suggested that compared to using antiplatelet therapy alone, the incidence of END was significantly reduced when patients were treated with Argatroban and antiplatelet therapy, while the mRS score significantly improved. At the same time, there was no increase in the risk and mortality of intracranial hemorrhage and other bleeding sites after the use of Argatroban. Overall, Argatroban has significant value in treating AIS.

## Data availability statement

The original contributions presented in the study are included in the article/Supplementary material, further inquiries can be directed to the corresponding authors.

## Author contributions

YC: Writing – review & editing. CL: Writing – review & editing. SL: Writing – review & editing. MM: Writing – review & editing. HL: Writing – review & editing.
