# Supplementary material for: Efficacy and safety of Argatroban in patients with acute ischemic stroke: a systematic review and meta-analysis
Source: Front Neurol. 2024 Feb 19;15:1364895. doi: 10.3389/fneur.2024.1364895 (PMC10909846; doi:10.3389/fneur.2024.1364895)
Supplement: Supplementary file 1 [file Table_1.DOCX]

**Table S1.** Search strategy

| **PubMed** | |
| --- | --- |
| #1 | Cerebrovascular Disorders[MeSH Terms] |
| #2 | stroke*[Title/Abstract] OR poststroke[Title/Abstract] OR apoplex*[Title/Abstract] OR cerebral vasc*[Title/Abstract] OR brain vasc*[Title/Abstract] OR cerebrovasc*[Title/Abstract] OR cva*[Title/Abstract] |
| #3 | (brain[Title/Abstract] OR cerebr*[Title/Abstract] OR cerebell*[Title/Abstract] OR vertebrobasil*[Title/Abstract] OR hemispher*[Title/Abstract] OR intracran*[Title/Abstract] OR intracerebral[Title/Abstract] OR infratentorial[Title/Abstract] OR supratentorial[Title/Abstract] OR middle cerebral artery[Title/Abstract] OR MCA*[Title/Abstract] OR anterior circulation[Title/Abstract] OR posterior circulation[Title/Abstract] OR basilar artery[Title/Abstract] OR vertebral artery[Title/Abstract] OR space‐occupying[Title/Abstract]) AND (ischaemi*[Title/Abstract] OR ischemi*[Title/Abstract] OR infarct*[Title/Abstract] OR thrombo*[Title/Abstract] OR emboli*[Title/Abstract] OR occlus*[Title/Abstract] OR hypoxi*[Title/Abstract]) |
| #4 | #1 or #2 or #3 |
| #5 | argatroban[Title/Abstract] |
| #6 | #4 and #5 |
| **Cochrane Library** | |
| #1 | MeSH descriptor: [Cerebrovascular Disorders] explode all trees |
| #2 | (stroke* or poststroke or apoplex* or cerebral vasc* or brain vasc* or cerebrovasc* or cva* or SAH):ti,ab,kw (Word variations have been searched) |
| #3 | ((brain or cerebr* or cerebell* or vertebrobasil* or hemispher* or intracran* or intracerebral or infratentorial or supratentorial or middle cerebral artery or MCA* or anterior circulation or posterior circulation or basilar artery or vertebral artery or space‐occupying) AND (ischaemi* or ischemi* or infarct* or thrombo* or emboli* or occlus* or hypoxi*)):ti,ab,kw |
| #4 | #1 or #2 or #3 |
| #5 | (argatroban):ti,ab,kw |
| #6 | #4 and #5 |
| **Embase** | |
| #1 | 'cerebrovascular disease'/exp |
| #2 | stroke*:ab,ti OR poststroke:ab,ti OR apoplex*:ab,ti OR 'cerebral vasc*':ab,ti OR 'brain vasc*':ab,ti OR cerebrovasc*:ab,ti OR cva*:ab,ti |
| #3 | (brain:ab,ti OR cerebr*:ab,ti OR cerebell*:ab,ti OR vertebrobasil*:ab,ti OR hemispher*:ab,ti OR intracran*:ab,ti OR intracerebral:ab,ti OR infratentorial:ab,ti OR supratentorial:ab,ti OR 'middle cerebral artery':ab,ti OR mca*:ab,ti OR 'anterior circulation':ab,ti OR 'posterior circulation':ab,ti OR 'basilar artery':ab,ti OR 'vertebral artery':ab,ti OR space鈥恛ccupying:ab,ti) AND (ischaemi*:ab,ti OR ischemi*:ab,ti OR infarct*:ab,ti OR thrombo*:ab,ti OR emboli*:ab,ti OR occlus*:ab,ti OR hypoxi*:ab,ti) |
| #4 | #1 OR #2 OR #3 |
| #5 | argatroban:ab,ti |
| #6 | #4 AND #5 |


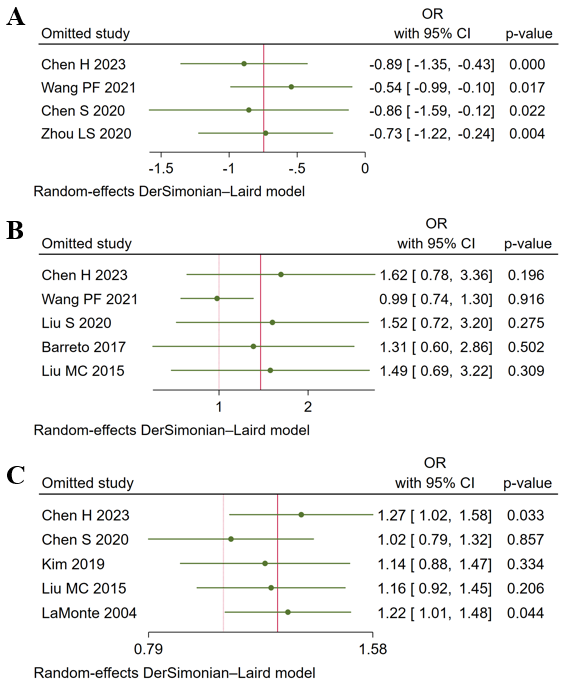


**Figure S1** Results of sensitivity analysis for (A) END, (B) mRS score of 0-1 (C) mRS score of 0-2 at 90 days by leave-one-out method


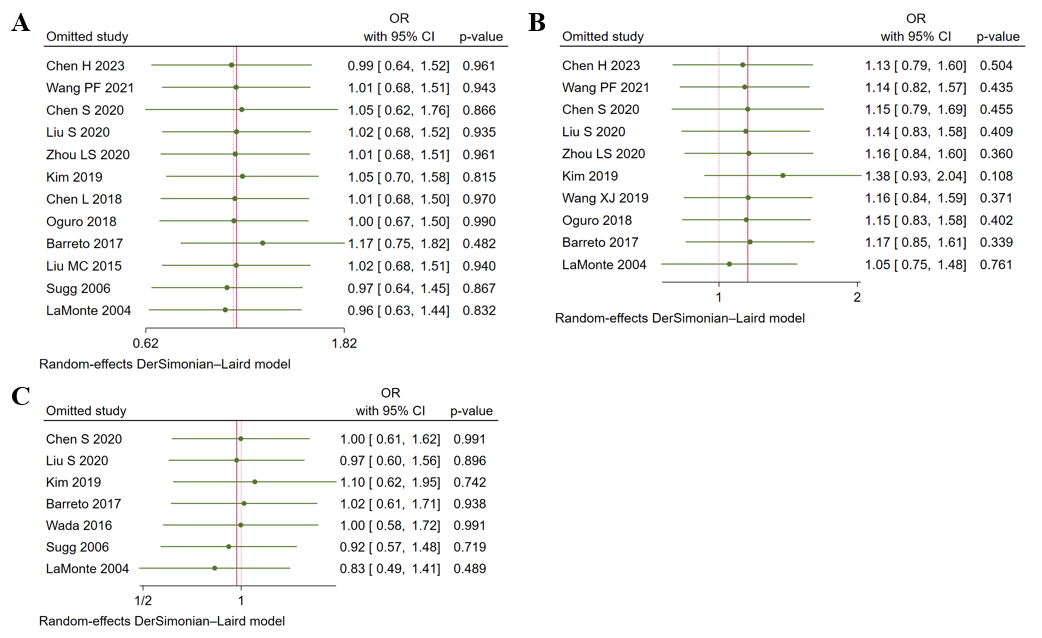


**Figure S2** Results of sensitivity analysis for adverse events by leave-one-out method. (A) intracranial hemorrhage; (B) major extracranial bleeding; (C) mortality.
